# Pitting Corrosion Behaviour of New Corrosion-Resistant Reinforcement Bars in Chloride-Containing Concrete Pore Solution

**DOI:** 10.3390/ma10080903

**Published:** 2017-08-04

**Authors:** Jin-yang Jiang, Yao Liu, Hong-yan Chu, Danqian Wang, Han Ma, Wei Sun

**Affiliations:** 1 School of Materials Science and Engineering, Southeast University, Nanjing 211189, China; liuyao0629@126.com (Y.L.); chuhongyan87@126.com (H.-y.C.); wonderbaba@126.com (D.W.); sunwei@seu.edu.cn (W.S.); 2 Jiangsu Key Laboratory of Construction Materials, Nanjing 211189, China; 3 Research Institute of Jiangsu Shasteel Iron and Steel, Zhangjiagang 215625, China; mahan-iris@shasteel.cn

**Keywords:** corrosion-resistant alloy steel bars, simulated concrete pore solution, digital holographic microscope, pitting corrosion

## Abstract

In this study, the pitting behaviour of a new corrosion-resistant alloy steel (CR) is compared to that of low-carbon steel (LC) in a simulated concrete pore solution with a chloride concentration of 5 mol/L. The electrochemical behaviour of the bars was characterised using linear polarisation resistance (LPR) and electrochemical impedance spectroscopy (EIS). The pitting profiles were detected by reflective digital holographic microscopy (DHM), scanning electron microscopy (SEM), and the chemical components produced in the pitting process were analysed by X-ray energy dispersive spectroscopy (EDS). The results show that the CR bars have a higher resistance to pitting corrosion than the LC bars. This is primarily because of the periodic occurrence of metastable pitting during pitting development. Compared to the pitting process in the LC bars, the pitting depth grows slowly in the CR bars, which greatly reduces the risk of pitting. The possible reason for this result is that the capability of the CR bars to heal the passivation film helps to restore the metastable pits to the passivation state.

## 1. Introduction

Pitting corrosion is one of the most widely occurring forms of localised corrosion in metal. It usually occurs in a series of corrosive environments where chloride is the most common corrosive ion [1,2]. Pitting generally occurs on the metal surface, and may cause perforation or stress corrosion. The pits are nucleated on a microscopic scale and are covered by the corrosion product; therefore, pitting is one of the more destructive and undetectable forms of corrosion in metals [3].

There are many factors that affect pitting corrosion. Bautista et al. [4] investigated the corrosion behavior of different kinds of steel bars subjected to various aggressive conditions. Major factors included the composition of the steel bars and the external conditions, such as the chemical composition of the environment and the pH. Pitting corrosion in metal occurs in three stages [5]: pitting nucleation, metastable pitting, and steady-state pitting. In recent years, the development of electrochemical technology has provided new methods for pitting corrosion research. Williams [6] found a higher anode current density at the boundary of inclusions using a scanning electrochemical microscope (SECM). Vuillemin et al. [7] observed the current ripple around the pits with the scanning vibrating electrode technique (SVET). Ameri et al. [8] studied the pitting corrosion behaviour of 316 stainless steel in a sodium chloride solution via zeta sets and constant potential polarisation. They found that maintaining the stability of the surface potential of the pitting product is important to the stable development of pitting corrosion. In addition to electrochemical measurements, Kocijan et al. [9] and Natishan et al. [10] conducted several studies on the surface features of pitting corrosion; however, the interaction between chloride ions and passivation films remains unclear. Ernst and Newman [11] experimentally studied two-dimensional (2D) growth at the edge of a stainless steel sheet, and proposed a semi-quantitative model for the lace-like pit cover. Laycock et al. [12] proposed 2D models to predict pitting corrosion growth under the influence of surface roughness, concentration of chloride in solution, and potential. Their results were in good agreement with experiments. Vignal et al. [13] studied the electrochemical behaviour of pitting in stainless steel using secondary ion mass spectrometry (SIMS), X-ray photoelectron spectroscopy (XPS), and SVET techniques. The experimental results were consistent with those of a numerical simulation. At present, many studies have investigated the electrochemical process and topography profiles of pitting corrosion, but the analysis of pitting morphology is limited to 2D plane effects. The three-dimensional (3D) development process of pitting is yet to be investigated.

In this study, the pitting behaviour of low-carbon steel (LC) is compared to that of a new corrosion-resistant alloy steel (CR) in a simulated concrete pore solution with a chloride concentration of 5 mol/L. The electrochemical behaviour of the steel bars is characterised using electrochemical measurements. The 2D morphological changes in the pitting are observed using scanning electron microscopy (SEM). The 3D morphology of the pits is analysed using reflective digital holographic microscopy (DHM).

## 2. Experiment

### 2.1. Raw Materials

Comparative studies are conducted using new Cr10Mo1 corrosion-resistant alloy steel (CR) bars and low-carbon steel (LC) bars. The specific composition of the steel is shown in Table 1.

There are no standards for the selection of components for the simulated concrete pore solutions. The primary influencing factors affecting corrosion are the chemical composition of the steel, the solution contents, and the pH [14]. As reported in the literature, a simulated solution can be roughly divided into one of two categories; i.e., it is either a highly alkaline solution or a saturated calcium hydroxide solution. Most highly alkaline solutions contain K^+^ and Na^+^ ions, and their pH values are greater than 13. The pH of the saturated calcium hydroxide solutions is generally between 12.5 and 12.6. Studies have shown that compared to the saturated calcium hydroxide solution, the high-alkaline solution is more similar to the actual concrete pore solution [15] Therefore, the simulated concrete pore solution used in this study is a highly alkaline solution with a pH of 13.6. In addition, 0.003 mol/L CaSO_4_ was also added to the highly alkaline solution, so as to make the highly alkaline solution more similar to the actual concrete pore solution. The etching solution used in this study is obtained by adding a 5 mol/L (5 M) sodium chloride solution to the above-mentioned simulated concrete pore solution. In order to accelerate the corrosion of the CR steel, a high concentration of chloride ion in the simulated pore solution was used in this paper. Details are listed in Table 2. The etching solution is prepared with vacuum filtration, using a 2.5 μm filter to ensure that the solution is free from suspensions.

### 2.2. Measurement Methods

#### 2.2.1. Electrochemical Measurements

The size of the steel bars used for testing was Φ20 mm × 10 mm (diameter is 20 mm, length is 10 mm). The bottoms of the bars were used as the testing surfaces. Each test surface was polished stepwise with increasing grades (#200, #600, #1000, and #2000) of SiC sandpaper, and the polishing time was about 5 min for each kind of sandpaper. After washing with deionised water, the surface was polished to a mirror finish with 0.25 μm diamond polishing liquid. The bars were then cleaned, and the surface impurities were removed with alcohol. The samples were dried with a hair dryer and immediately placed into the etching tank.

The bars were immersed in a simulated concrete pore solution with a 13.6 pH and 5 M chloride concentration for 0 h, 24 h, 60 h, 120 h, 200 h, and 240 h. The electrochemical test was then performed with the PARSTAT 4000 electrochemical workstation. A three-electrode measurement system was used; i.e., the reinforced bars were set as the working electrode, the saturated calomel electrode was set as the reference electrode, and the platinum electrode was the auxiliary electrode. The test was performed at room temperature (25 ± 1 °C). All electrochemical tests were performed after the open potentials working electrode was stabilised.

##### Linear Polarisation Resistance Method

The linear polarisation resistance method is a common method for measuring the corrosion rate of steel bars [16]. In this study, the polarisation resistance of the bar surface is measured by adding a small polarisation potential near the corrosion potential. The corrosion current [17] is then calculated as follows:

(1) $$i_{corr}=B/R_{p}$$

where *R_p_* is the polarisation resistance (kΩ·cm^2^), *i_corr_* is the corrosion current density (μA/cm^2^), and *B* is the Stern-Geary [18] constant (mV). For reinforcement bars in concrete, if the bars are corroded, *B* is 26 mV; in the passivated state, *B* is 52 mV. The range of the linear polarisation resistance is ±10 mV vs. E_corr_ at a scanning speed of 10 mV/min.

##### Electrochemical Impedance Spectroscopy

Electrochemical impedance spectroscopy (EIS) is a frequency domain-based nondestructive test method, and it has been widely used to characterise the corrosion behaviour of steel bars [19,20]. A sinusoidal voltage excitation signal with a disturbance amplitude of 10 mV is used. The frequency range is 10^4^~10^−2^ Hz. The kinetic information in the electrochemical process is obtained by measuring the periodic response of the corrosion system.

#### 2.2.2. Micro-Measurements

Steel bars measuring 10 mm × 10 mm × 5 mm were used in these tests. The surface was polished stepwise with increasing grades (#200, #600, #1000, and #2000) of SiC sandpaper. After washing with deionised water, the surface was polished to a mirror finish with a 0.25 μm diamond polishing liquid. The bars were then cleaned, and the surface impurities were removed with alcohol. The samples were dried with a hair dryer and immediately immersed into a simulated 5 M concrete pore solution for 0 h, 24 h, 60 h, 100 h, 120 h, and 200 h.

To perform the micro-measurements, the samples were removed from the corrosive solution, the sample surface was washed with deionised water, and the sample was placed in anhydrous ethanol for 30 min for ultrasonic cleaning. The sample was removed from the solution and rinsed with anhydrous ethanol, ultrasonically cleaned for another 30 min, then dried with a hair dryer. The samples were placed onto a watch glass and dried in an oven.

##### Reflective Digital Holographic Microscopy

A Lyncee Tec (Lyncee Tec SA, Lausanne, Vaud, Switzerland) digital holographic microscope, a type of reflective digital holographic microscope, with a view field of 5 mm, was used in this experiment. The resolution along the *x*- and *y*-axes is >300 nm, and the resolution along the *z*-axis is >0.6 nm. The surfaces of the samples etched for different durations was viewed with a lens of 20× magnification. The 3D morphology of the pitting corrosion in the same area was measured after the sample endured various times of corrosive exposure. The pitting process was analysed microscopically.

##### Scanning Electron Microscopy (SEM)

The scanning electron microscope used in this experiment is a field emission scanning electron microscope (SIRION-100, FEI, Eindhoven, Noord-Brabant, The Netherlands). During the test, the surface profiles of the samples were viewed under 10,000× and 30,000× magnifications. Elemental analyses were performed both inside and outside of the pitting corrosion areas.

## 3. Experiments and Discussion

### 3.1. Electrochemical Test

#### 3.1.1. Linear Polarisation Curve

**Figure 1:**
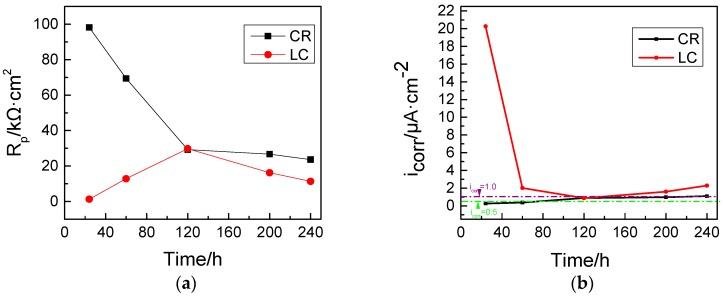
Linear polarisation resistance (**a**) and corrosion current density (**b**) of two types of steel bars at different corrosive exposure times.

The data in Figure 1 show that, after 24 h, the corrosion current density of the LC bars is as high as 20 μA·cm^−2^; however, for the corrosion resistant steel bars, it is only approximately 0.2 μA·cm^−2^. This indicates the corrosion of both types of bars after being immersed in the corrosive solution for 24 h. The corrosion current density of the LC bars reaches 2.3 μA·cm^−2^ after 240 h, and continues to increase. On the contrary, the corrosion current density of the CR bars increases slowly, only reaching approximately 1.0 μA·cm^−2^ after 240 h, and remains stable afterwards. The decrease of *i_corr_* is because the material in solution contributed to creating a protective coating on the surface of the LC bars, while the gradual increase of *i_corr_* for the LC bars after 120 h was due to the pitting corrosion of the LC bars. It should be noted that the current density numbers cannot be used to study the corrosion rate of the CR bars when pitting is seen.

#### 3.1.2. Electrochemical Impedance Spectroscopy

**Figure 2:**
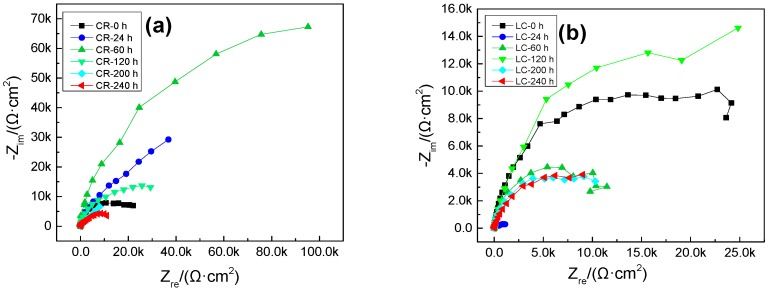
Nyquist plot at different corrosive duration times: (**a**) the CR bars; and (**b**) the LC bars.

The data in Figure 2a show that before the samples have soaked for 60 h, the radius of the capacitive loop of the CR steel increases gradually, indicating that the corrosion resistance increases with soaking time. After 24 h of soaking, the high-frequency band is still a capacitive loop. The low frequency band, however, changes into a straight line with a slope of 45°, i.e., the Warburg impedance with characteristics of diffusion. This indicates that the corrosion of the system is controlled by the diffusion of the oxygen process. After immersion for 60 h, the radius of the capacitive loop in the CR steel bars begins to decrease gradually, and thus the corrosion resistance also decreases gradually. All of the above demonstrate that the passivation film is formed in the CR steel bars even when the concentration of corrosive ions reaches the critical concentration of chloride ions [21]. The pitting is nucleated after approximately 24 h of immersion, and the pitting corrosion starts to develop after 60 h of immersion.

In the case of the LC steel bars, the capacitive loop is extremely small after 24 h immersion in chloride as shown in Figure 2b. This suggests the immediate corrosion of the LC bar in the chloride solution. Thus, it can be deduced that the passive film of LC bars cannot stay stable in chloride or even that there is no passive film formed on the LC bar. After 24 h of immersion, the capacitive loop becomes larger as the exposure time increases. This may be caused by the continuous development of pitting corrosion. The corrosion products continue to increase attaching to the interface between the steel bars and the concrete pore solution. Their presence hinders the ion transport between the steel bars and the pore solution. Compared to the CR steel bars, the polarisation resistance of the LC bars is lower, indicating that the corrosion resistance of the LC steel bars is significantly lower than that of the CR steel in a chloride environment.

### 3.2. Micro Measurements and Analyses

#### 3.2.1. Digital Holographic Microscopy Analysis

**Figure 3:**
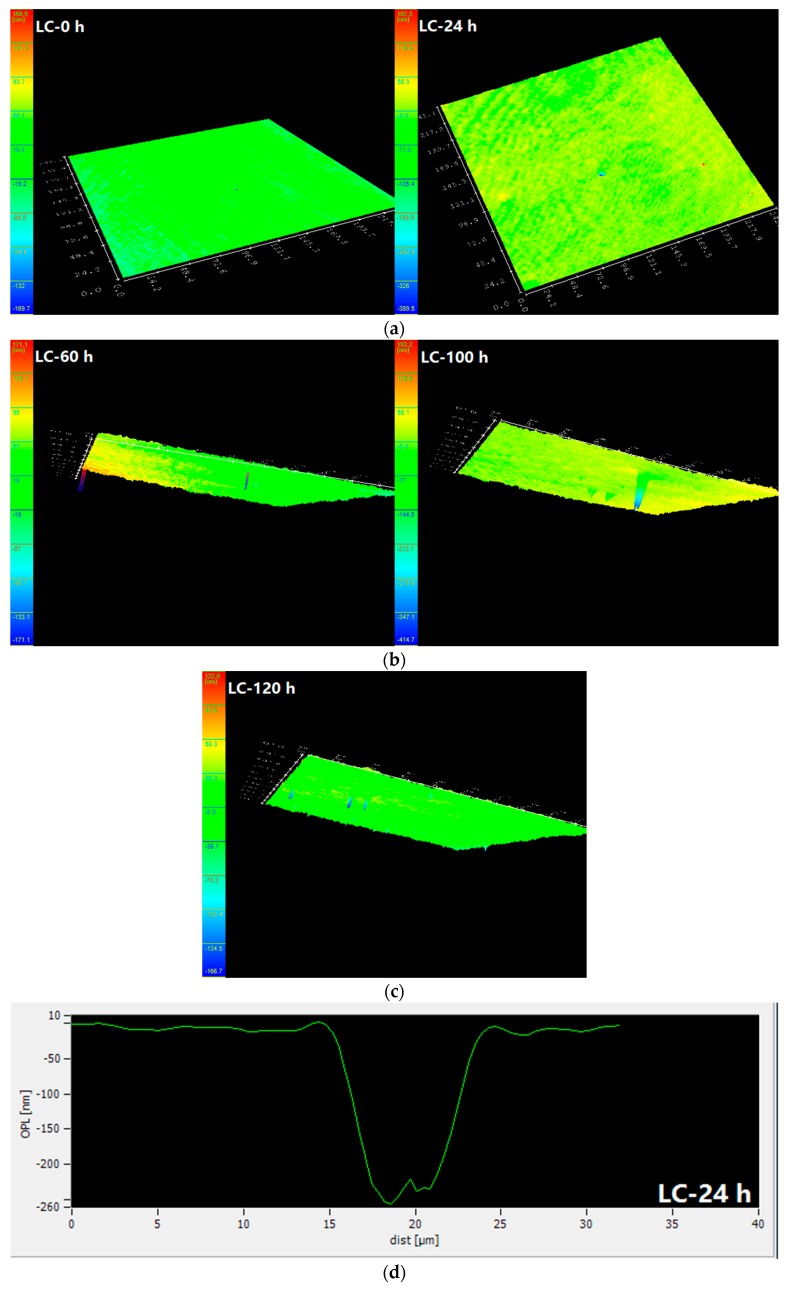

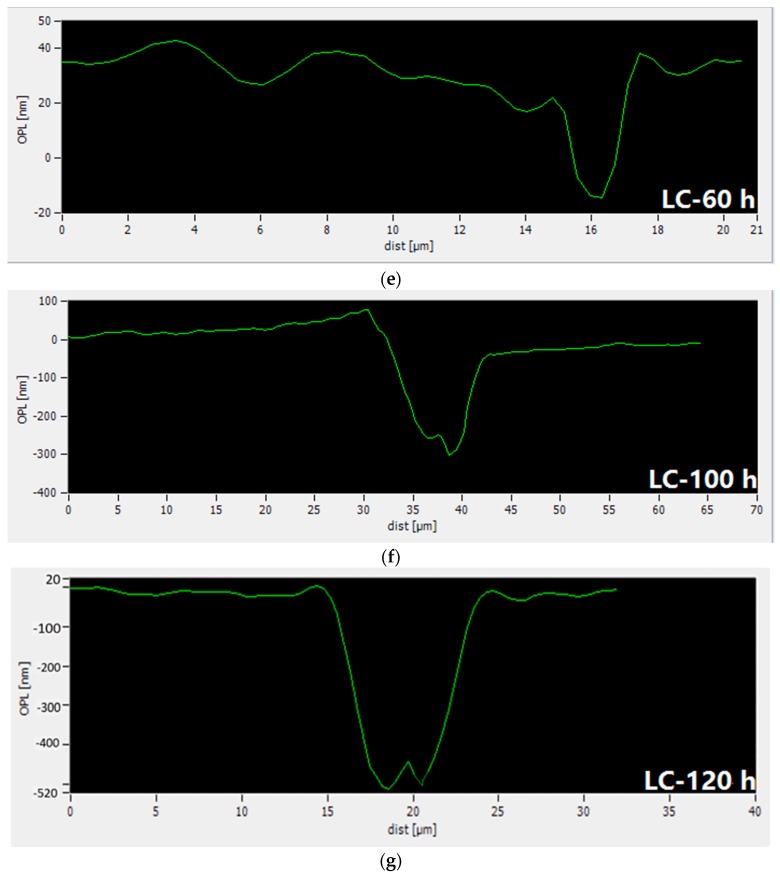
Surface morphology and pitting depth in carbon steel bars at different corrosive exposure times. (**a**) Surface morphology for LC at 0 and 24 h; (**b**) Surface morphology for LC at 60 and 100 h; (**c**) Surface morphology for LC at 120 h; (**d**) Pitting depth for LC at 24 h; (**e**) Pitting depth for LC at 60 h; (**f**) Pitting depth for LC at 100 h; and (**g**) Pitting depth for LC at 120 h. (OPL: Optical path length).

Figure 3 shows the surface morphology and the depth of the pits in carbon steel bars at different corrosive exposure times. It shows that at the start of the test (0 h), the steel surface is smooth and stable; no surface irregularities are present. After soaking for 24 h in the corrosive solution, the steel surface shows obvious signs of roughness. There are some small peaks on the surface, and the depth of the pits is 50 nm. After 60 h of immersion, the surface is further roughened. The maximum depth of the pits is 300 nm. As the soaking duration reaches 100 h, the pit is visible (as shown in Figure 3). Its depth is measured at 350 nm. There are two peaks at the bottom of the pit, indicating that new pitting corrosion is nucleated, further damaging the bottom of the pits. After soaking for 120 h, pits are prominently visible. The depth of the pits is approximately 500 nm, which indicates that, at this time, pitting corrosion is developing along the vertical direction.

**Figure 4:**
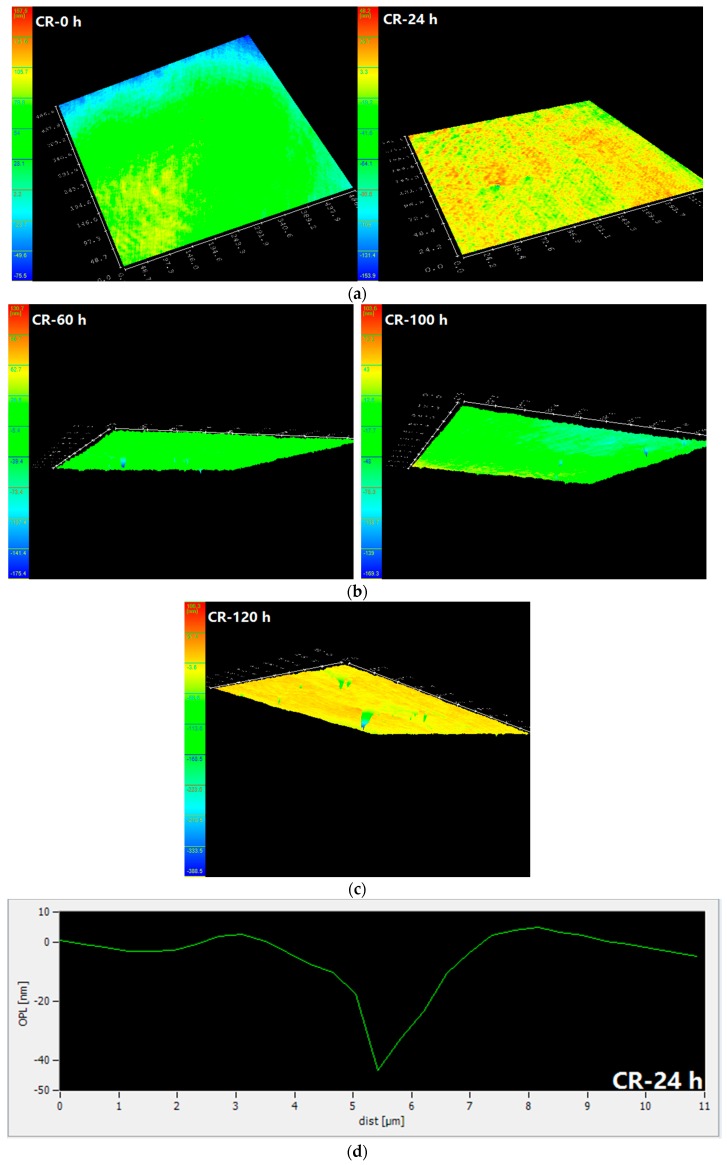

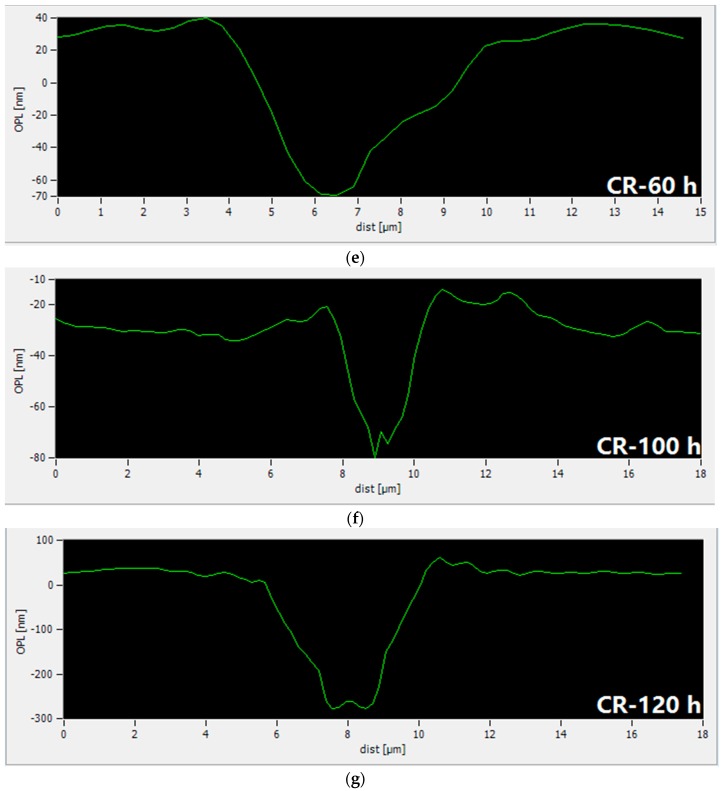
Surface morphology and height and depth of pits in corrosion resistant alloy steel bars at different corrosive exposure times. (**a**) Surface morphology for CR at 0 and 24 h; (**b**) Surface morphology for CR at 60 and 100 h; (**c**) Surface morphology for CR at 120 h; (**d**) Height and depth of pits for CR at 24 h; (**e**) Height and depth of pits for CR at 60 h; (**f**) Height and depth of pits for CR at 100 h; and (**g**) Height and depth of pits for CR at 120 h.

Figure 4 shows the surface topography, height, and depth of the pits in the corrosion resistant steel bars at different corrosive exposure times. The pitting depth of the CR bar increases with immersion time, which is similar to the LC bar. However, the pitting depth of the CR bar is much smaller than that of the LC bar before 100 h of immersion, while the pitting depth of the CR bar sharply increases to 300 nm after 120 h of immersion. In addition, it should be noted that the pitting width of the CR bar is significantly smaller than that of the LC bar during the whole immersion period.

These results, combined with the results of the electrochemical test, show that the CR bars are passivated at 0 h. The pitting corrosion is nucleated at approximately 24 h, and starts to develop at 60 h. According to the literature [22], under the attack of corrosive chloride ions, the passivation film possibly breaks around the inclusions in the CR bars, thus creating pits with curvature radii of dozens of nanometres. The reason for this phenomenon is that these inclusions are favourable locations for pitting initiation [23].

#### 3.2.2. Scanning Electron Microscopy Analysis

**Figure 5:**
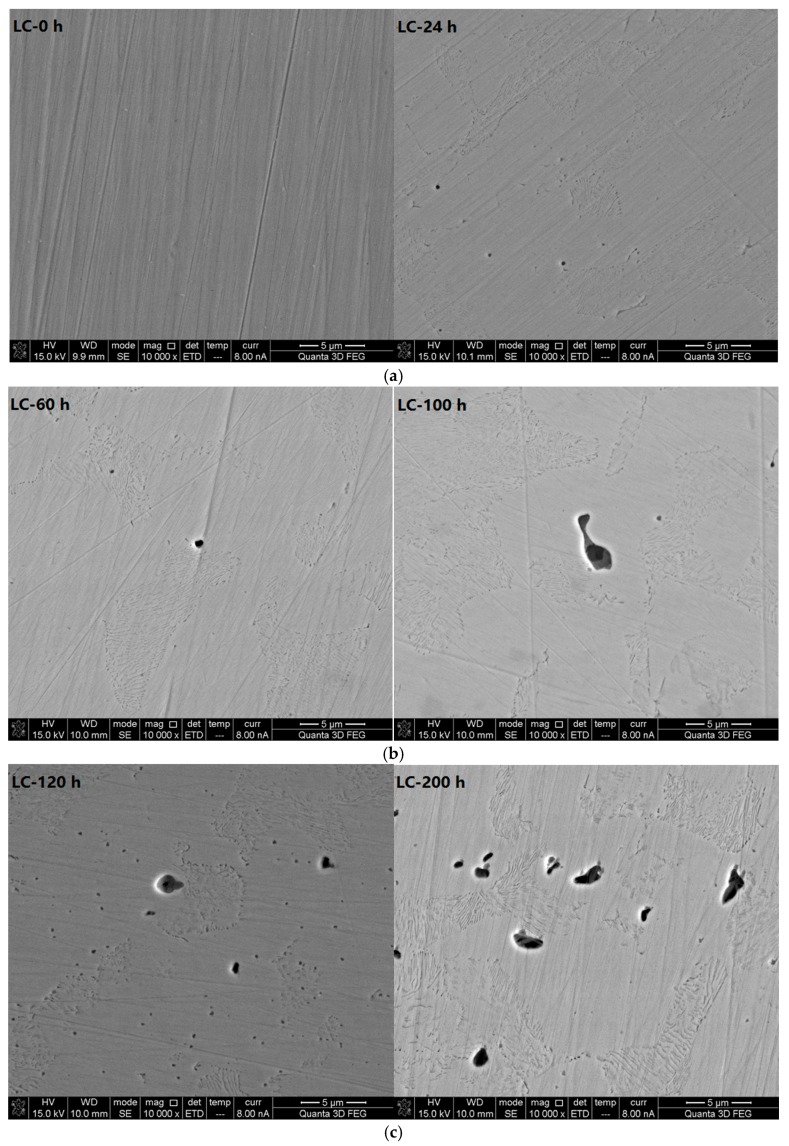
Scanning electron microscope photos (10,000× magnification) of carbon steel bars at different corrosive exposure times. (**a**) Micrograph of LC at 0 and 24 h; (**b**) Micrograph of LC at 60 and 100 h; and (**c**) Micrograph of LC at 120 and 200 h.

Figure 5 shows photos taken with a scanning electron microscope (10,000× magnification) of the LC samples at different corrosion immersion times. The data show that when the test starts (0 h), the surface is flat without corrosion pits. After immersion for 24 h, round pits of nano-scale diameters appear distributed on the surface of the LC bars. After soaking in corrosive solutions for 60 h, regular circular holes of 1 μm in diameter appear on the surface; the vast majority of the uniformly distributed holes of nano-scale diameter formed after 24 h of immersion and disappeared after 60 h of immersion. After immersion for 100 h, large areas of irregular pitting corrosion appear on the surface. A large pit is created by the connection and further growth of two adjacent pits. The bottoms of the pits have stepped contours. After immersion for 120 h, in addition to some large holes, small holes of nano-scale diameters are distributed around the large holes. After immersion for 200 h, large holes of irregular shapes appear on the surface. The percentage of large holes is high. The data show that after soaking for 120 h in the corrosive solution, pitting corrosion has developed across the surfaces of the LC samples. The pitting corrosion kind for the CR steel bar was steady-state pitting corrosion. Additionally, the reinforcement bar generally experiences a high corrosion rate, which is consistent with the electrochemical test results.

**Figure 6:**
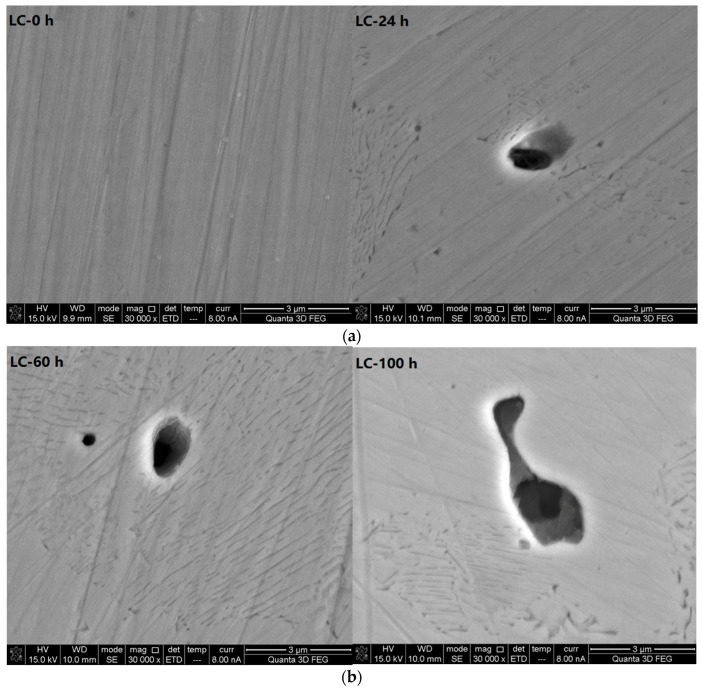

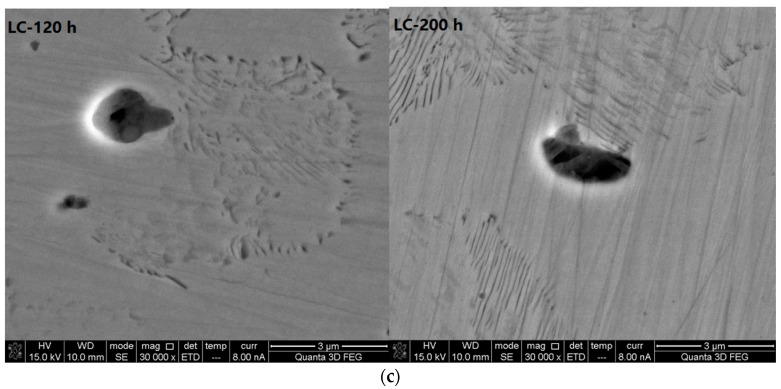
Scanning electron microscope photos (30,000× magnification) of carbon steel bars at different corrosive exposure times. (**a**) Micrograph of LC at 0 and 24 h; (**b**) Micrograph of LC at 60 and 100 h; and (**c**) Micrograph of LC at 120 and 200 h.

The SEM photos (30,000× magnification) of the LC bars at different corrosive exposure times (see Figure 6) show that the pitting corrosion starts as stepped pits, then develops layer by layer until reaching the bottom of the pit.

**Figure 7:**
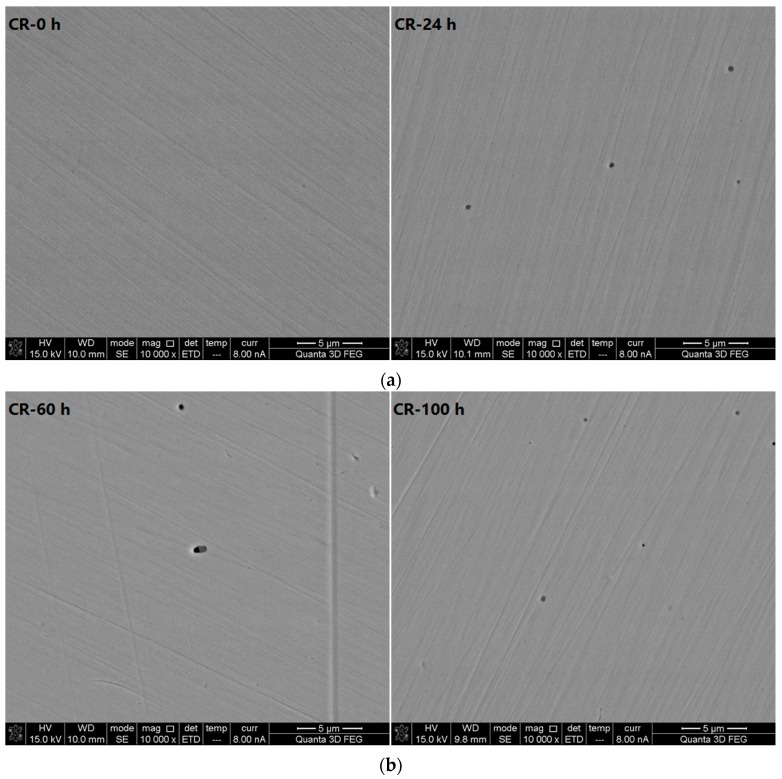

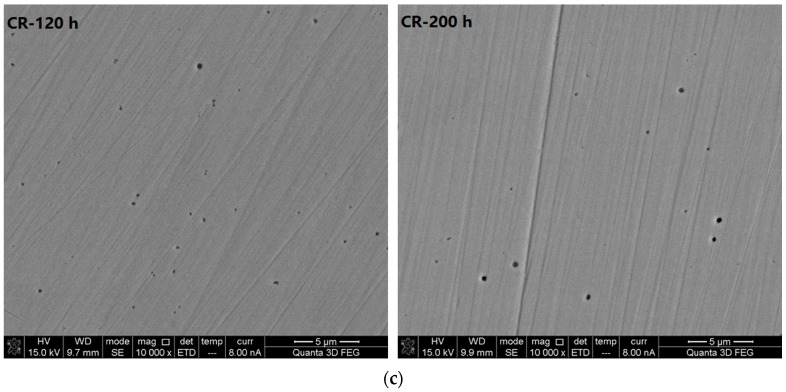
Scanning electron microscope photos (10,000× magnification) of corrosion-resistant alloy steel bars at different corrosive exposure times. (**a**) Micrograph of CR at 0 and 24 h; (**b**) Micrograph of CR at 60 and 100 h; and (**c**) Micrograph of CR at 120 and 200 h.

The scanning electron microscope photos (10,000× magnification) in Figure 7 show the CR samples at different corrosive exposure times. The data show that in the beginning (0 h), the surface is smooth compared to that of the LC bars, and there is no pitting corrosion. After immersion for 24 h, a small number of corrosion pits of nano-scale diameters appear on the surface, mostly in regular, round shapes. After immersion for 60 h, individual large pits appear; the largest diameter is approximately 1 μm. However, the number of small pits, i.e., pits of nano-scale diameter, in the areas surrounding the large pits decreases. This result suggests that the nano-scale pits are possibly re-passivation, resulting in the remaining good corrosion resistance of the CR bar, which is consistent with the electrochemical results. After immersion for 100 h, individual large corrosion pits appear on the surface. Some small pits, i.e., pits of nano-scale diameter, also appear. After immersion for 120 h, some micron-scale corrosion pits appear, with some more widely distributed smaller nano-scale pits. After immersion for 200 h, the size of the pits remains the same; however, the number of nano-scale pits is less than it was at 120 h. Pistorius et al. [24] showed that the development of pitting corrosion includes pitting nuclei, metastable pitting corrosion, steady-state pitting corrosion, and growth stoppage, or the re-passivation of pitting corrosion pits known as metastable pitting corrosion. Metastable pitting corrosion occurs before the formation of steady-state pitting corrosion. However, the mechanism of the formation of metastable pitting corrosion is still unclear [25].

The results show that in the simulated concrete pore solution with a Cl^−^ concentration of 5 mol/L, before the immersion duration reaches 200 h, the maximum diameter of the corrosion pits in the CR samples is 1 μm. The steel bar passes through the following periodic process under chloride attack. The passivation film is roughened and the pits are nucleated, which creates nano-scale corrosion pits. Some of the nano-scale pitting corrosion pits are unstable, exhibiting metastable pitting corrosion. Some portion of the pits is then passivated and disappears, while the rest of the nano-scale pits develop further into micron-scale pits. A dynamic balance is maintained when the pits grow to approximately 1 μm. During the entire periodic process, in the initial period, i.e., during the immersion period of 24 h, 60 h and 100 h, there are few pitting nuclei or metastable corrosion pits. This is because, by this time, the surface of the CR steel is still passivated because it is not prone to pitting corrosion. During the period of 100 h, 120 h and 200 h, the quantity of metastable pitting corrosion increases since the accumulation of chloride ions on the surface of the CR bar reaches a certain level. This is consistent with the results from the macro electrochemical test. The corrosion rate of the CR bars is high after they have been immersed for 120 h. The corrosion rate of the CR steel decreases at 120 h. The adsorption of chloride ions during pitting nucleation damages the passivation film, resulting in the formation of nano-scale pores. Under certain conditions, the nano-scale pores continue to grow, and the pitting corrosion enters the pitting development stage, forming micron-scale corrosion pits.

**Figure 8:**
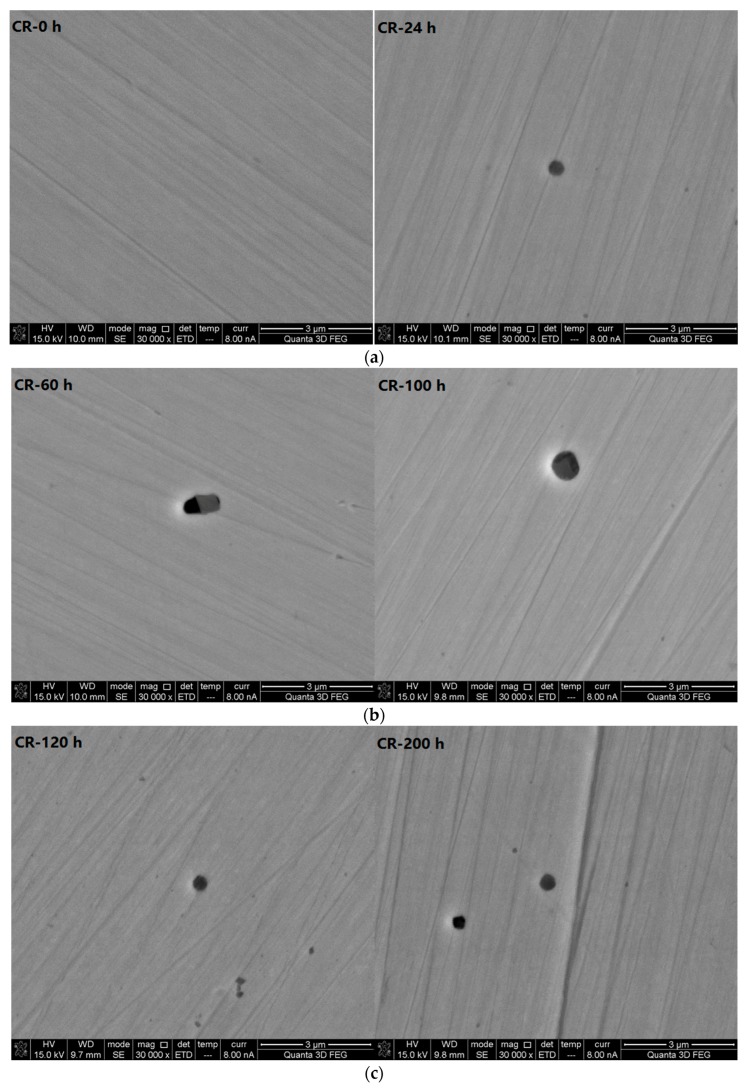
Scanning electron microscope photos (30,000× magnification) of corrosion-resistant alloy steel bars at different corrosive exposure times. (**a**) Micrograph of CR at 0 and 24 h; (**b**) Micrograph of CR at 60 and 100 h; and (**c**) Micrograph of CR at 120 and 200 h.

The scanning electron microscope photos (30,000× magnification) of the CR bars at different corrosive exposure times (see Figure 8) show that, in CR steel, the shape of the pitting corrosion pits is primarily a regular circle. Each individual pit is independent of the others. Pits generally extend to the bottom. This is different from the evolving pattern of pitting corrosion in the LC bars.

#### 3.2.3. Analysis of the Pitting Corrosion Process in Corrosion-Resistant Alloy Steel Bars

**Figure 9:**
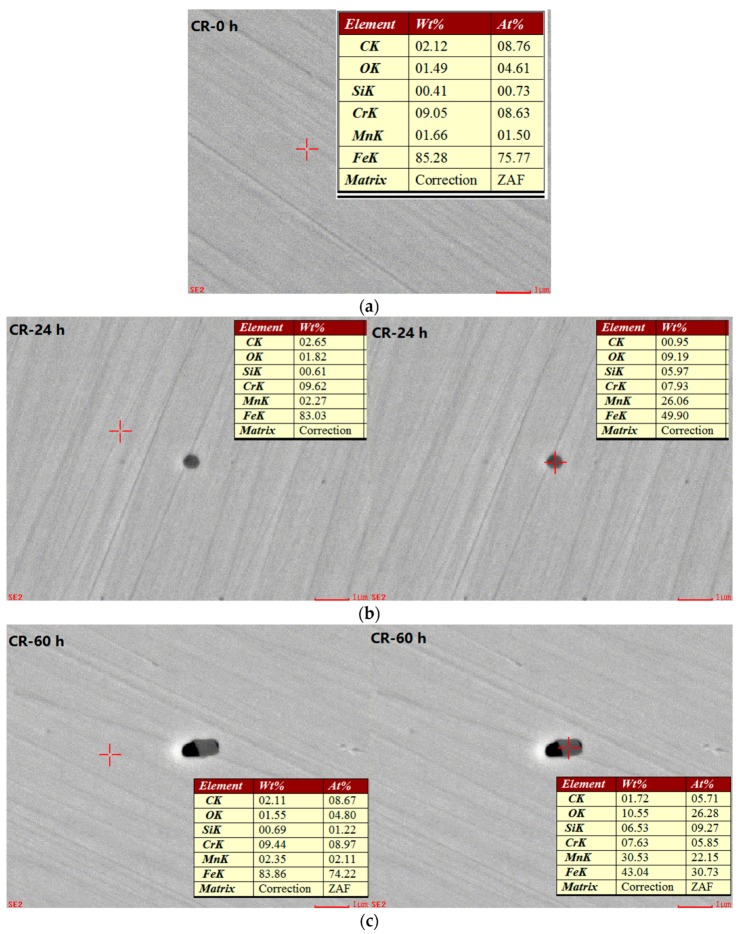

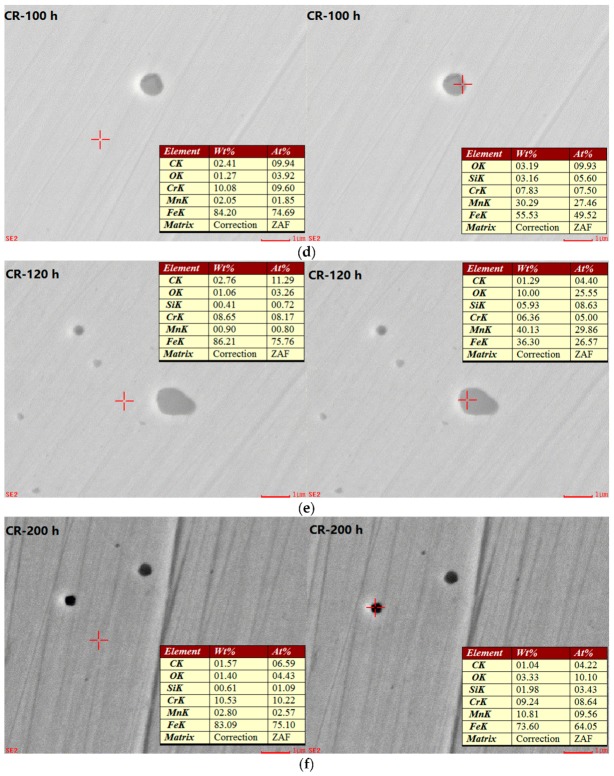
Energy dispersive spectroscopy (EDS) photos of corrosion-resistant alloy reinforcement bars at different corrosion exposure times. (**a**) EDS result of CR at 0 h; (**b**) EDS result of CR at 24 h; (**c**) EDS result of CR at 60 h; (**d**) EDS result of CR at 100 h; (**e**) EDS result of CR at 120 h; and (**f**) EDS result of CR at 200 h.

To analyse the effect of the chemical components on the pitting corrosion development process in CR bars, the chemical compositions inside and outside of the corrosion pits versus the immersion duration are analysed using an energy dispersive spectroscopy (EDS) analysis. It should be noted that the correction method is ZAF in EDS analysis. The results are shown in Figure 9.

**Figure 10:**
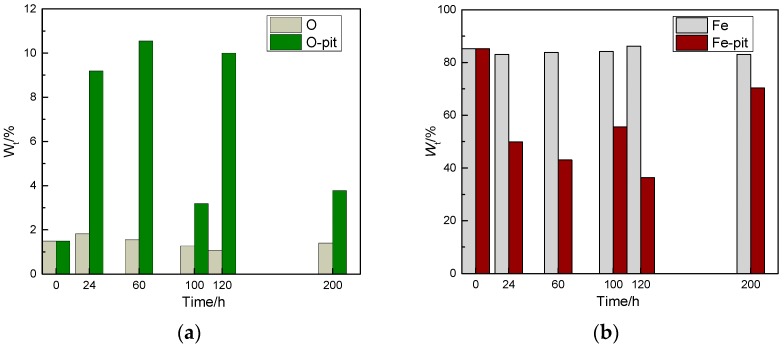
Analysis of the chemical elements inside and outside of the corrosion pits at different corrosion exposure times in corrosion-resistant alloy steel bars. (O-pit represents the O content inside the pits, Fe-pit represents the Fe content inside the pits). (**a**) O element contained in inside and outside of the corrosion pits; and (**b**) Fe element contained in inside and outside of the corrosion pits.

**Figure 11:**
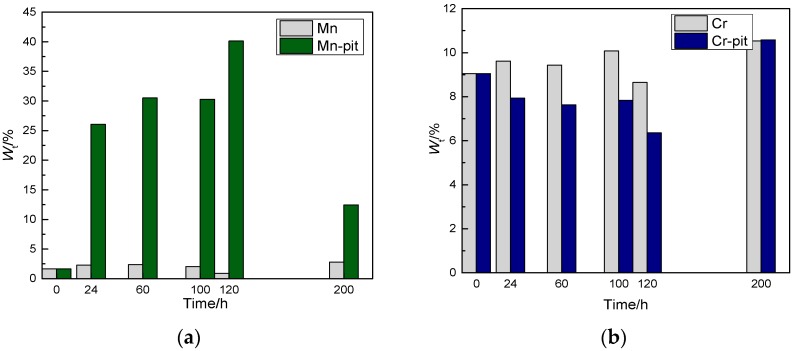
Contents of chemical elements inside and outside of the corrosion pits at different corrosion exposure times in corrosion-resistant alloy steel bars. (Mn-pit represents Mn content inside the pits, Cr-pit represents the Cr content inside the pits). (**a**) Mn element contained in inside and outside of the corrosion pits; and (**b**) Cr element contained in inside and outside of the corrosion pits.

A determination of the amount of the elements O, Fe, Cr, and Mn, both inside and outside of the corrosion pits in CR bars at different corrosion exposure times, is obtained by analysing the results shown in Figure 9. The details are shown in Figure 10 and Figure 11.

The data in Figure 10 show that there is more O inside the corrosion pits than outside of the pits, whereas there is less Fe inside the pits than outside of the pits. This is in agreement with the results of Figueira et al. [26]. The reason for the oxygen distribution is that the corrosion products generated inside the corrosion pits contain oxygen, whilst the area outside of the pits is in the passive state. Hence, the oxygen content inside the pits is high. In addition, the corrosion reaction occurs inside the pits. Therefore, the iron is oxidised into corrosive products, which are found inside the pits.

The data in Figure 11 show that the quantity of Mn inside the corrosion pits is much higher than that outside of the pits. This is because the pitting corrosion occurs at the Mn inclusions during the nucleation period [27], resulting in pitting corrosion. Generally, the pitting corrosion formed in these locations will continue to develop into steady-state pitting. The quantity of Cr inside the pits is low for the following reasons. On one hand, according to the literature [28], Cr is involved in the formation of passive films, thereby resulting in a film that consists of an inner layer that contains Cr–Fe oxides and an outer layer that contains Fe oxides, whose thickness presents a slight increase as the content of Cr increases. Cr can effectively enhance the CR steel’s resistance to corrosion. Therefore, pitting occurs in locations where the Cr content is low. On the other hand, in the electrochemical corrosion process involved during the development of pitting corrosion, the chromium (Cr) ions inside the pits are not dissolved as the iron ion is, but rather, they remain along the internal walls of the pits. Hence, the Cr content inside the pits is low. As demonstrated by the pitting corrosion model, the stable development of the corrosion pits is closely related to the chemical elements at the bottom of the pits. Iron, chromium, and molybdenum are dissolved by oxidation, and the metal ions are created inside the corrosion pits. These ions are hydrolysed, and then form hydroxide and hydroxyl chloride complexes [29].

A microscopic analysis shows that the steady-state corrosion pitting in CR bars has a generally circular shape, and the pitting develops longitudinally in the direction of gravity. The present study shows that pitting is caused by local damage of the passivation film. The damaged passivation film is the anode, while the intact passivation film is the cathode. Once the pits are formed, the surface of the CR bar in the area of the pitting is in a localised active state (the potential is low). The anode dissolves in the pits, generating the elements Fe^2+^, Cr^3+^, etc. Most of the steel surface outside the pits is still in the passivated state (with high potential). The cathode reduction reaction takes place on the outer surfaces of the adjacent pit via O_2_ + 2H_2_O + 4e^−^ → 4OH^−^. Therefore, the activation/passivation battery cell composed of a small anode and large cathode is formed inside and outside the pits, which accelerates the pitting corrosion. Simultaneously, in strongly alkaline conditions, there is a large quantity of OH^−^ ions in the solution. Once Fe^2+^ ions precipitate from the pits, they will react with OH^−^ to produce Fe(OH)_2_. As the solution is not deoxygenated, the generated Fe(OH)_2_ rapidly reacts with the dissolved O_2_ in the solution to form Fe(OH)_3_. The compound Fe(OH)_3_ decomposes rapidly to form Fe_2_O_3_. In addition, because of the high Cr content in the CR bars, Cr ions will participate in the reaction, generating Cr_2_O_3_. In this process, the oxygen diffusion is the controlling factor. The corrosion products generated are quickly adsorbed around the pits, creating an occlusion zone. The oxygen is depleted inside the pits, while outside the pits, the oxygen is rich. This creates an oxygen concentration battery cell. The main reason for the slow pitting of the CR bar during the first 60 h is possibly the inhabitation of Cr around the MnS inclusion. The redox corrosion reaction is difficult to produce in these areas, and leads to the pitting of the lower width, which inhibits the transport of ions into pits. Thus, the corrosion reaction in the pit is reduced. Some of the pits are repassive when the passive reaction rate is higher than the corrosion reaction rate, while other pits slowly continue to develop.

**Figure 12:**
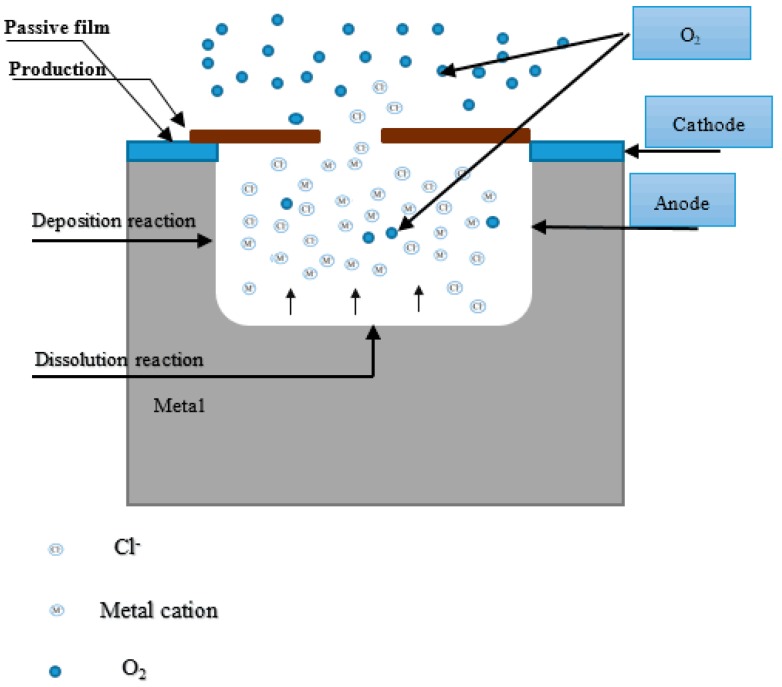
Development of pitting corrosion pits exposed to corrosive media in corrosion-resistant alloy steel bars.

The above analysis shows that, during pitting corrosion, the activation/passivation battery, and the oxygen concentration by the battery, occur inside and outside of the corrosion pits in the CR bars. The development of pitting corrosion in the CR bars is presented in Figure 12. As shown in Figure 12, coupled with the gravity, the pit has a deep excavating capacity. During the pitting corrosion development, the dissolved chromium ions are not all diffused and hydrolysed as the Fe^2+^ ions are. On the contrary, some of the chromium ions are re-deposited onto the internal walls of the pits. After discharging, the Cr ions deposit inside the pits, creating a surface layer of high chromium content. Therefore, a dissolution reaction occurs primarily at the bottom of the pit, and pitting corrosion develops a more regular shape as it develops vertically. In addition, a large area outside of the corrosion pit is in the passivated cathode state, further accelerating the metal ionisation process inside the pits. However, because the corrosion products are deposited in the openings of the pits, the passivation areas around the pit openings are cathodically protected by electrons released from the anodic process. This greatly suppresses the corrosion around the pits. For this reason, the pitting corrosion in the CR bars appears as the distribution of a single pit.

## 4. Conclusions

In this study, the pitting corrosion behaviours of LC bars and CR bars exposed to a simulated concrete pore solution are compared. Macroscopic electrochemical and microscopic analyses show that CR steel has better resistance to pitting corrosion than LC steel. The primary reasons for the better performance of the CR steel are as follows:

(1) CR steel has a high passivation capability. Compared with the polished LC samples that are immersed into the corrosive solution, a passivation film is formed on the surfaces of the CR bars. This film hinders the initiation of pitting. In LC steel, however, pitting corrosion occurs immediately, resulting in steady-state pitting corrosion.
(2) The corrosion pits in CR bars are mostly individually developed shallow pits of circular shape. The corrosion pits in the LC samples, however, are large, irregular pits, created by the connection of adjacent pits. The corrosion pits in CR bars are regularly shaped micro-nanometre-scale pits. The pits are primarily caused by Cl^−^ absorption that compromises the passivation film. Compared to the rapid development of corrosion pits and the large pit size in LC steel samples, the corrosion pits in the CR bars develop slowly. Hence, the CR bars have great advantages over LC bars in controlling hazards caused by perforation corrosion, which is the result of uncontrolled pitting corrosion development.
(3) The formation of metastable pitting corrosion greatly alleviates the pitting growth rate in CR bars. Pitting corrosion develops immediately into steady-state pitting after pitting nucleation in LC bars; however, the CR bars have a significant metastable pitting stage that occurs periodically. This is because the corrosion pits in the CR bars are shallow and the corrosion current density is small. Hence, closed pits are easily formed. For all of these reasons, the pitting corrosion at the metastable pitting stage is passivated and then disappears.

## Figures and Tables

**Table 1:** Chemical composition of the experimental steel (percentage).

Chemical Composition	C	Si	Mn	Cr	Cu	Ni	Al	Mo
LC	0.22	0.53	1.44	-	-	-	-	-
CR	0.01	0.487	1.49	10.36	-	-	-	1.162

LC, low-carbon steel; CR, corrosion-resistant alloy steel.

**Table 2:** Corrosive soaking solutions for Steel.

Corrosive Solution	KOH	NaOH	Ca(OH)_2_	NaCl
Concentration (mol/L)	0.6	0.2	0.03	5
